# Epidemiological trends of HSV-1 and HSV-2 Central Nervous System Infections: A Retrospective Analysis from 2018 to 2023 from Saudi Arabia

**DOI:** 10.25122/jml-2024-0377

**Published:** 2025-01

**Authors:** Abdulaziz Alhazmi, Amal Gharawi, Khalid Alameer, Bandar Abuageelah, Ehab Hakami, Taif Zogel, Abdullah Almalki, Ebhar Magrashi, Wafa Alharbi, Ramis Manni, Atheer Buayti, Ali Qasem, Tareq Khawaji, Moayad Haddad, Nabil Dhayhi

**Affiliations:** 1 Department of Basic Medical Sciences, Faculty of Medicine, Jazan University, Jazan, Saudi Arabia; 2 Health Sciences Research Center, Jazan University, Jazan, Saudi Arabia; 3 Department of Medicine and Surgery, Faculty of Medicine, Jazan University, Jazan, Saudi Arabia; 4 Department of Medicine and Surgery, Batterjee Medical College, Aseer, Saudi Arabia; 5 Prince Mohammed Bin Nasser Hospital, Jazan Health Cluster, Jazan, Saudi Arabia; 6 Regional Laboratory, Jazan Health Cluster, Jazan, Saudi Arabia; 7 Laboratory, King Fahad Central Hospital, Jazan Health Cluster, Jazan, Saudi Arabia; 8 Department of Pediatric Infectious Diseases, King Fahad Central Hospital, Jazan Health Cluster, Jazan, Saudi Arabia

**Keywords:** Herpes Simplex Virus, CNS infections, HSV-1, HSV-2, PCR, Saudi Arabia, Jazan

## Abstract

Herpes Simplex Virus (HSV) types 1 and 2 are significant etiologies to central nervous system (CNS) infections, leading to potential severe neurological consequences. Despite their global impact, our region's data on the epidemiology of HSV CNS infections remains limited. This study assessed the epidemiology and diagnostic trends of HSV-1 and HSV-2 CNS infections in a tertiary hospital in Southwestern Saudi Arabia from 2018 to 2023. A retrospective study was conducted using data from cerebrospinal fluid (CSF) samples collected from patients with suspected CNS infection between 2018 and 2023. Polymerase Chain Reaction (PCR) results for detecting HSV-1 and HSV-2, performed as part of routine clinical diagnostics, were analyzed. Data on patient demographics, specimen collection times (including months and years), laboratory processing times, and seasonal trends were collected and analyzed using descriptive statistics and univariate analysis. Of the 280 samples, 11.0% were positive for HSV, with 10.0% positive for HSV-1 and 1.0% for HSV-2. Most HSV-positive cases were found in patients aged ≥51 years (27.0%). Peak detection occurred in 2020, with seasonal spikes in May and September. The turnaround time significantly varied, with the shortest laboratory turnaround time for PCR results recorded in 2020 (35.54 hours), while the longest was in 2021 (167.38 hours). This study reports an important burden of HSV-related CNS infections in Southwestern Saudi Arabia, indicating the importance of timely diagnosis through PCR testing. Our findings highlight the need for improved diagnostic workflows and enhanced epidemiological surveillance. Future research should explore broader regional and clinical data related to HSV CNS infection epidemiology.

## INTRODUCTION

Herpes Simplex Virus (HSV) types 1 and 2 are significant causes of viral infections, often leading to severe neurological outcomes, including viral meningitis and encephalitis [1]. HSV infections of the central nervous system (CNS) are particularly concerning in immunocompromised individuals and the elderly due to their association with high morbidity and mortality rates. HSV is classified into two different viruses, HSV-1 and HSV-2, belonging to the herpesvirus family of DNA viruses [1]. HSV-1 predominantly causes orofacial infections, while HSV-2 is more commonly associated with genital infections. However, both viruses can establish lifelong latency and reactivate under certain conditions, leading to recurrent infections [1]. Notably, both HSV-1 and HSV-2 can cause viral meningitis and, more commonly, viral encephalitis, making timely diagnosis and management crucial to improving patient outcomes [2].

Globally, HSV is recognized as a significant cause of CNS infections [3]. A systematic review and meta-analysis of 71 studies found the overall prevalence of HSV encephalitis among patients tested for viral CNS infections to be 8.0% and HSV meningitis to be 4.0% [3]. The review also reported age differences in HSV prevalence, with adult patients showing a higher prevalence of HSV encephalitis (12.0%) and meningitis (9.0%) compared to pediatric populations. These findings highlight the considerable burden of HSV-related CNS infections worldwide, indicating the need for accurate diagnosis and prompt treatment to reduce morbidity and mortality. The high rate of missed cases due to inadequate use of highly sensitive diagnostic tools also emphasizes the importance of improving diagnostic practices globally to better manage CNS infections caused by HSV [3].

To accurately identify HSV in suspected cases of CNS infections, polymerase chain reaction (PCR) testing of cerebrospinal fluid (CSF) is considered the gold standard. This method provides rapid and specific detection of HSV, aiding clinicians in differentiating between various causes of CNS infections [4]. Despite significant advancements in molecular diagnostic techniques, including syndromic methods, challenges persist in all phases of laboratory testing, particularly in the pre-analytical and analytical stages [3-5].

In Saudi Arabia, there is a lack of comprehensive data on the epidemiology of HSV in the context of CNS infections, likely due to the limited availability of large-scale studies, underreporting, and inconsistent access to molecular diagnostic tools across healthcare facilities in the regions [4-7]. Thus, this study aims to address this gap by conducting a retrospective analysis of HSV-1 and HSV-2 infections identified through PCR testing in CSF samples collected at a tertiary hospital in the Southwestern region of Saudi Arabia between 2018 and 2023. By evaluating temporal trends, demographic characteristics, and laboratory processing times, this study provides insights into the burden of HSV-associated neurological infections over six years and informs healthcare professionals about the epidemiological patterns of HSV in Saudi Arabia. Additionally, this study aimed to evaluate the demographic distribution of HSV-positive cases, assess the turnaround times for PCR diagnostics, and explore potential seasonal and yearly trends in HSV-related CNS infections.

## MATERIAL AND METHODS

### Study design and setting

A retrospective analysis was conducted at a tertiary hospital in Southwestern Saudi Arabia, using data from CSF samples from patients suspected of CNS infections. The samples were submitted to the microbiology laboratory between January 2018 and December 2023. This hospital serves as the designated referral center for the region and the only facility within the area equipped for neurosurgeries, with a capacity of 500 beds.

### Inclusion and exclusion criteria

The study included all CSF samples from adult patients (≥18 years) who tested positive for HSV-1 or HSV-2 between January 2018 and December 2023. Exclusion criteria included pediatric patients (<18 years), patients with other types of infectious meningitis, as well as those with repeated, missing data or incomplete medical records. Children were excluded from the study to focus on the adult population with different epidemiological characteristics and disease outcomes.

### Data collection

Data was collected from the medical records system and verified against local data from the microbiology laboratory. Collected information included patient demographics (age), CSF collection date, the causative virus (HSV-1 or HSV-2), the time from sample collection to laboratory receipt (in hours), and turnaround time (in hours). Data was organized using Microsoft Excel (version 2023, Microsoft Corporation, Redmond, WA, USA). Confidentiality and accuracy of the data were prioritized throughout the data collection process.

### CSF collection

At our center, CSF samples are routinely collected via lumbar puncture under aseptic conditions and immediately processed upon arrival at the laboratory. Testing for HSV-1 and HSV-2 was performed using the Artus HSV-1/2 RG PCR Kit (QIAGEN GmbH), following the manufacturer’s instructions. Viral DNA was extracted from CSF using the EZ1 DSP Virus Kit (QIAGEN GmbH). The real-time PCR assay targeted a 154 bp region specific to both HSV-1 and HSV-2, with results detected through the fluorescence channels Cycling Green and Cycling Orange, respectively. The analytical sensitivity of the test was 0.12 copies/µl for HSV-1 and 0.16 copies/µl for HSV-2, with a 95.0% probability of detection based on the manufacturer's data. Internal controls were included in every run to monitor for PCR inhibition, and positive controls for both HSV-1 and HSV-2 were used to ensure accuracy. All reagents were handled according to storage guidelines to prevent contamination.

### Data quality assurance

Patient data was extracted into a Microsoft Excel spreadsheet to ensure data integrity. Data entries were validated by cross-referencing with original medical records, and any discrepancies were resolved through joint re-examination of the source documents. Patients with missing records were excluded from the analysis.

### Statistical analysis

Data was organized in a tabular format and analyzed descriptively, with means calculated and frequency tables generated. Statistical analyses were performed using IBM SPSS (version 25, Armonk, NY: IBM Corp.). Univariate analysis using t-test and chi-square assessed individual variables, with statistical significance defined as *P* ≤ 0.05.

## RESULTS

This retrospective study analyzed 285 clinical specimens obtained from the medical record system and confirmed with the record system at the microbiology laboratory between 2018 and 2023. Five CSF samples were excluded due to missing data.

As illustrated in Table 1, most samples were from elderly patients, with 78 samples (27.0%) from individuals aged ≥ 51, followed by 75 (27.0%) from those aged 18-28. PCR testing indicated that 10.0% were positive for HSV-1 and 1.0% for HSV-2. Most samples were received in 2020 (22.0%) and the fewest in 2023 (13.0%). The time between specimen collection and receipt in the laboratory showed that most samples took 36 hours or more, accounting for 41.0% (115), while 39.0% (108) took between 12 and 23.9 hours. The turnaround time for reporting PCR results to clinicians was 72 hours or more for 30.0% (85) of the samples.

**Table 1:** Descriptive analysis for the included data between 2018–2023

Variable	*n* = 280		
Age group (years)	18–28	75	27.0%
	29–39	74	26.0%
	40–50	53	19.0%
	≥ 51	78	28.0%
Viral PCR	Negative	248	89.0%
	HSV-1	29	10.0%
	HSV-2	3	1.0%
Year	2018	38	14.0%
	2019	50	18.0%
	2020	61	22.0%
	2021	43	15.0%
	2022	52	19.0%
	2023	36	13.0%
Time from sample collection to receipt in the laboratory (hours)	0–11.9 h	43	15.0%
	12–23.9 h	108	39.0%
	24 –35.9 h	14	5.0%
	≥ 36 h	115	41.0%
Turnaround time (hours)	0–23.9 h	79	28.0%
	24–47.9 h	84	30.0%
	48–71.9 h	32	11.0%
	≥ 72 h	85	30.0%

This retrospective study compared variables between HSV PCR-negative and positive CSF samples from 2018-2023. Most reported cases were among individuals aged 29-39 years (34.0%), followed by elderly patients aged 51 or older, with 28.0% of HSV-1 and HSV-2 cases. No statistically significant differences were detected between the age groups, months, or years of specimen collection. Similarly, mean times from collection to laboratory receipt and turnaround time had no significant differences between groups. Table 2 provides further details regarding the study’s variables categorized based on the results of the viral PCR test.

**Table 2:** Variables categorized based on results of viral PCR test (*n* = 280)

Variable		Negative		HSV-1&2		*P* value
		*n* = 248	%	*n* = 32	%	
Age groups (years)	18–28	71	29.0%	4	13.0%	0.229
	29–39	63	25.0%	11	34.0%	
	40–50	45	18.0%	8	25.0%	
	≥ 51	69	28.0%	9	28.0%	
Month	JAN	31	13.0%	2	6.0%	0.543
	FEB	25	10.0%	4	13.0%	
	MAR	31	13.0%	2	6.0%	
	APR	9	4.0%	2	6.0%	
	MAY	21	8.0%	5	16.0%	
	JUN	14	6.0%	1	3.0%	
	JUL	17	7.0%	3	9.0%	
	AUG	21	8.0%	2	6.0%	
	SEP	14	6.0%	5	16.0%	
	OCT	29	12.0%	2	6.0%	
	NOV	18	7.0%	2	6.0%	
	DEC	18	7.0%	2	6.0%	
Year	2018	30	12.0%	8	25.0%	0.276
	2019	44	18.0%	6	19.0%	
	2020	54	22.0%	7	22.0%	
	2021	38	15.0%	5	16.0%	
	2022	47	19.0%	5	16.0%	
	2023	35	14.0%	1	3.0%	
Time from sample collection to receipt in the laboratory (hours)	0– 11.9 h	37	15.0%	6	19.0%	0.814
	12– 23.9 h	98	40.0%	10	31.0%	
	24–35.9 h	12	5.0%	2	6.0%	
	≥ 36 h	101	41.0%	14	44.0%	
Turnaround time (hours)	0–23.9 h	71	29.0%	8	25.0%	0.268
	24–47.9 h	76	31.0%	8	25.0%	
	48–71.9 h	25	10.0%	7	22.0%	
	≥ 72 h	76	31.0%	9	28.0%	

Table 3 demonstrates the time from specimen collection to receipt in the laboratory and the turnaround time of results in the laboratory across the study years. Samples collected during 2023 show the shortest transport time to the lab with a mean of 24.45 hours (± 20.25 SD), while those collected in 2021 had the longest transport time to the lab at 45.43 hours (±39.43 SD). Regarding turnaround time, 2020 had the shortest mean time of 35.54 hours (± 69.55 SD), while 2021 had the longest with 167.38 hours (± 424.44 SD).

**Table 3:** Time from sample collection to receipt in the laboratory and turnaround time in hours categorized by years of study (2018-2023)

Year	Average time from sample collection to receipt in the laboratory (hours)	Average turnaround time (hours)
2018	29.76 ± 32.83	50.67 ± 44.12
2019	41.13 ± 25.41	72.46 ± 165.10
2020	37.37 ± 33.30	35.54 ± 69.55
2021	45.43 ± 39.43	167.38 ± 424.44
2022	40.34 ± 38.99	53.63 ± 42.48
2023	24.45 ± 20.25	65.42 ± 131.51

## DISCUSSION

The findings from this study provide valuable insights into the epidemiology of HSV-1 and HSV-2 infections detected in CSF samples in a tertiary hospital setting in Southwestern Saudi Arabia. The results, based on data collected between 2018 and 2023, reveal important patterns regarding the prevalence of HSV in CNS infections, as well as the demographic characteristics of affected patients and some valuable data regarding the pre-analytical and analytical phases of HSV PCR. These findings may add to the limited knowledge of HSV epidemiology in this region and have several clinical and public health implications [4,5].

In our study, 11.0% of CSF samples tested positive for both HSV-1 and HSV-2, with 10.0% being positive for HSV-1 and 1.0% for HSV-2. These findings are consistent with global trends, where HSV-1 is more commonly associated with CNS infections than HSV-2, particularly in cases of meningitis and encephalitis [3]. Rohani *et al*. recently published a systematic review and meta-analysis of 71 studies, reporting an overall prevalence of 8.0% for HSV encephalitis and 4.0% for HSV meningitis, with notable regional variations [3]. In Saudi Arabia, a study found a 1.0% prevalence of HSV-2 in CSF samples, specifically in cases of aseptic meningitis, using a multiplex real-time PCR kit [6]. Another recent study (2024) reported that HSV was responsible for 36.0% of meningitis and encephalitis cases compared to other infectious causes in a 7-year, single-center study [7]. In Qatar, a neighboring country, a retrospective study from 2015 to 2018 found that 1.5% of cases were caused by HSV-1 and 2.0% by HSV-2, both identified as causative agents of viral meningitis using the same multiplex real-time PCR technique [8]. The higher detection rate of HSV-1 in our study, compared to HSV-2, may reflect the dominance of HSV-1 in causing CNS infections in adults, particularly in the Middle East, where early acquisition of HSV-1 is common due to its high seroprevalence [9]. Additionally, variations in the reported prevalence of HSV-1 and HSV-2 across studies may be attributed to differences in methodology, regional viral transmission patterns, diagnostic techniques, study populations, and the availability of advanced molecular testing, emphasizing the need for standardized approaches to better understand the regional and global burden of HSV-related CNS infections [3].

The temporal analysis of CSF samples revealed a seasonal and annual trend in HSV-related CNS infections. In this study, peak detection rates occurred in May and September, suggesting potential seasonal patterns in HSV reactivation or transmission [10-12]. This aligns with previous findings indicating seasonal variations in herpes viral infections, potentially driven by environmental factors like temperature, changes in immune responses, or increased social interactions during certain months, as observed with other members of the herpes family. However, other studies reported no seasonal variations patterns associated with herpes viral infections in general [10-12]. On the other hand, the annual trends show that 2018 and 2020 had the highest number of HSV-positive cases, with a decline observed in 2021 and 2022. The increase in 2020 could be attributed to the increased diagnostic efforts during the COVID-19 pandemic, where patients presenting with neurological symptoms were broadly tested for viral pathogens, including HSV [13]. However, others suggest that HSV reactivation is frequent in patients with COVID-19 [14]. The rise in viral CNS infection detection during the pandemic due to comprehensive testing is expected, and the subsequent decline in 2021 and 2022 is notable and may be due to the reduced transmission of viral infections during the pandemic when social distancing and hygiene measures were in place [15].

One of the key findings in our study relates to the time from specimen collection to receipt in the laboratory and the turnaround time for reporting PCR results. The mean time between specimen collection and laboratory receipt was 37.14 hours, with significant variability across the study years. The shortest mean time was observed in 2023 (24.45 hours), while the longest was in 2021 (45.43 hours). This variability likely reflects logistical challenges, such as transportation delays and differences in laboratory capacity during the COVID-19 pandemic [16-18]. Turnaround time for PCR results also showed considerable variability, with the shortest mean time in 2020 (35.54 hours) and the longest in 2021 (167.38 hours). The extended turnaround times in 2021 may be attributable to the increased burden on healthcare systems during the peak of the pandemic, which affected laboratory resources and delayed diagnostic work-up [19,20]. Despite these challenges, the relatively short turnaround times in other years highlight the efficiency of molecular testing in providing rapid results, which is crucial for the timely management of CNS infections.

The results of this study have many important implications for laboratory and clinical practice as well as public health in Saudi Arabia. The relatively high prevalence of HSV in CSF samples indicates the need for clinicians to consider HSV as a potential cause of CNS infections, especially in adults and the elderly. Plus, early detection through PCR testing is crucial for timely antiviral treatment when indicated, improving outcomes and reducing mortality. The variability in lab processing times also necessitates more efficient diagnostic workflows, particularly in resource-limited settings. Improved epidemiological reports and access to advanced molecular diagnostic tools are essential to better manage viral CNS infections. However, the study bears some limitations. Its retrospective design may have introduced selection bias, as only patients who underwent PCR testing were included, possibly underestimating the true prevalence of HSV CNS infections. The single-center nature of the study limits its generalizability to other regions in Saudi Arabia. Furthermore, excluding five samples due to missing data may have influenced the results. Finally, the absence of clinical data related to patient outcomes and the lack of data on COVID-19 infection and potential co-infection with HSV further restrict the comprehensiveness of the analysis. Future studies should focus on larger sample sizes and more comprehensive data collection to better understand HSV epidemiology in the region.

## CONCLUSION

In conclusion, this study highlights the significant burden of HSV-related CNS infections and indicates the need for timely diagnosis. Efforts to improve diagnostic workflows, enhance epidemiological surveillance, and expand access to molecular diagnostic tools will be crucial in reducing the impact of HSV on public health in Saudi Arabia. Further research is needed to confirm these findings and explore potential seasonal and regional variations in HSV epidemiology.

## Data Availability

The data presented in this study are available upon request from the first author.
